# Experimental evaluation of protection and immunogenicity of *Streptococcus suis* bacterin-based vaccines formulated with different commercial adjuvants in weaned piglets

**DOI:** 10.1186/s13567-021-01004-x

**Published:** 2021-10-19

**Authors:** Milan R. Obradovic, Lorelei Corsaut, Dominic Dolbec, Marcelo Gottschalk, Mariela Segura

**Affiliations:** Research Group On Infectious Diseases in Production Animals (GREMIP) and Swine and Poultry Infectious Diseases Research Centre (CRIPA), Faculty of Veterinary Medicine, University of Montreal, 3200 Sicotte, Saint-Hyacinthe, QC J2S 2M2 Canada

**Keywords:** *Streptococcus suis*, swine, bacterin vaccines, adjuvants, antibodies, Alhydrogel®, Emulsigen®-D, Quil-A®, Montanide™

## Abstract

*Streptococcus suis* is an important swine pathogen responsible for economic losses to the swine industry worldwide. There is no effective commercial vaccine against *S. suis*. The use of autogenous (“bacterin”) vaccines to control *S. suis* outbreaks is a frequent preventive measure in the field, although scientific data on immunogenicity and reduction in mortality and morbidity are scarce. The goal of our study is to experimentally evaluate the immunogenicity and protective efficacy against homologous challenge in weaned piglets of a *S. suis* serotype 2 bacterin-based vaccine formulated with six different commercial adjuvants (Alhydrogel®, Emulsigen®-D, Quil-A®, Montanide™ ISA 206 VG, Montanide™ ISA 61 VG, and Montanide™ ISA 201 VG). The vaccine formulated with Montanide™ ISA 61 VG induced a significant increase in anti-*S. suis* antibodies, including both IgG1 and IgG2 subclasses, protected against mortality and significantly reduced morbidity and severity of clinical signs. Vaccines formulated with Montanide ISA 206 VG or Montanide ISA 201 VG also induced a significant increase in anti-*S. suis* antibodies and showed partial protection and reduction of clinical signs severity. Vaccines formulated with Alhydrogel®, Emulsigen®-D, or Quil-A® induced a low and IgG1-shifted antibody response and failed to protect vaccinated piglets against a homologous challenge. In conclusion, the type of adjuvant used in the vaccine formulation significantly influenced the immune response and efficacy of the vaccine against a homologous challenge.

**Supplementary Information:**

The online version contains supplementary material available at 10.1186/s13567-021-01004-x.

## Introduction

*Streptococcus suis* is a Gram-positive bacterium with 29 serotypes described based on the immunogenicity of its capsular polysaccharide (CPS) [1]. It causes disease in weaned and, occasionally, in suckling and grower piglets, with clinical signs of meningitis, arthritis, endocarditis, septicemia, and sudden death [2]. *S. suis* has a worldwide prevalence and it is estimated that 100% of pig farms are positive, since it is normal inhabitant of the upper respiratory tract [3, 4]. Serotypes 2 and 9 are by far the most prevalent in Europe. In North America, serotypes 1 to 9 and 14 are usually associated to disease, being serotypes 1/2 and 2 the most prevalent [5, 6]. *S. suis* is also a zoonosis, causing mainly meningitis and septic shock, with high importance in certain Asian countries where raw pig products are traditionally consumed [7, 8]. Antimicrobials are not only used to treat disease but also as prophylaxis/metaphylaxis to control *S. suis* in pig herds, although their use has been limited in some countries due to the increasing occurrence of antimicrobial resistance [9].

Prevention of clinical disease caused by *S. suis* is mainly based on the control of predisposal factors and the use of vaccines [10]. Since there are no universal efficacious commercial vaccines against *S. suis* infection, the use of autogenous vaccines (in general, bacterins) is widespread [11]. The production of the *S. suis* autogenous vaccine starts with the isolation of a specific bacterial strain(s) causing the problem in a particular farm; this bacterial isolate is then killed (generally with formalin) and formulated with a specific adjuvant [12]. The plethora of *S. suis* serotypes and strains, and a wide variety of adjuvants and vaccine production methods, make the evaluation of the efficacy of autogenous vaccines difficult, and often ambiguous data are acquired from field trials [11]. A recent field study showed that vaccination of piglets with a licensed autogenous vaccine composed of *S. suis* serotype 7 strain adjuvanted with oil-in-water adjuvant (confidential formulation) failed to induce an active immune response and clinical protection after vaccination of piglets [13]. In a vaccination study in piglets with an experimental *S. suis* serotype 2 bacterin, protection was recorded when formulated with a water-in-oil emulsion but not with Alhydrogel® [14]. Adjuvants are key components of a vaccine formulation and have the capacity to not only increase the vaccine-induced immune response but also modulate the type of this response and consequently the protection level obtained. Despite the importance of adjuvants, few studies have compared the effect of different adjuvants in the same experimental trial or at least under the same conditions [14, 15].

The goal of this study was to evaluate the influence of different commercial adjuvants included in a *S. suis* serotype 2 bacterin-vaccine formulation on the immunogenicity and protection against homologous challenge. This is the first controlled experimental study conducted to compare the effect of six different commercial adjuvants, widely used in animal vaccine production, on *S. suis* vaccine efficacy.

## Materials and methods

### Preparation of the bacterin

*Streptococcus suis* serotype 2 strain P1/7, a well-characterized virulent reference strain [16], was grown overnight onto 5% sheep blood agar plates at 37 °C, and isolated colonies were cultured in 5 mL of Todd–Hewitt broth (THB; Becton Dickinson, Mississauga, ON, Canada) for 8 h at 37 °C. Then, 450 μL of 1/1000 dilution of 8-h cultures were transferred into two volumes of 1.3 L of THB each and incubated for 16 h at 37 °C. Bacteria were centrifuged 10 000 × *g* for 40 min at 4 °C, pellets were re-suspended in 250 mL of sterile phosphate-buffered saline (PBS) and centrifuged 10 000 × *g* for 10 min at 4 °C. Two additional washing steps of the bacterial pellet with PBS were performed using the same centrifugation settings. After the final wash, the pellet was re-suspended in 250 mL of sterile PBS and the bacterial count was performed. To inactivate the bacteria, formaldehyde was added to the final concentration of 0.5% and incubated for 48 h at 4 °C. The mixture was screened for sterility and washed three times with sterile PBS, using the same centrifugation settings. The bacterin pellet was re-suspended in 250 mL of sterile PBS and thiomersal was added to a final concentration of 0.01% v/v. Each immunization dose contained the equivalent to 10^9^ CFU/mL.

### Formulation of *S. suis* bacterin with different adjuvants

Six commercial adjuvants were used to make different formulations of the vaccine in this study. Vaccine formulations consisted of formalin-inactivated 10^9^ CFU of *S. suis* serotype 2 strain P1/7 formulated with Alhydrogel®-2% (Croda, formerly known as Brenntag Biosector A/S, InvivoGen, San Diego, CA, USA) at a final concentration of 50% v/v (Group 1), Emulsigen®-D (MVP Adjuvants®, Phibro Animal Health Corporation, Teaneck, NJ, USA) at a final concentration of 20% v/v (Group 2), Quil-A® (Croda) at a final concentration of 0.3 mg/mL (Group 3), Montanide™ ISA 206 VG (Group 4), Montanide™ ISA 61 VG (Group 5), and Montanide™ ISA 201 ISA VG (Group 6) (SEPPIC, Fairfield, NJ, USA). All procedures for vaccine formulations with tested adjuvants were done according to the manufacturer’s protocols. Placebo controls (corresponding adjuvant only) were included in each group. The six different commercial adjuvants were used to make vaccine formulations using the same formalin-killed bacterial suspension of *S. suis* serotype 2 strain P1/7.

### Animals

This study was carried out in accordance with the recommendations of the guidelines and policies of the Canadian Council on Animal Care and the principles outlined in the Guide for the Care and Use of Laboratory Animals. The protocols and procedures were approved by the Animal Welfare Committee of the University of Montreal (protocol number Rech-2014). Recently weaned, three-week-old, Landrace/white mixed breed piglets were acquired from a commercial farm in Quebec, with no history of clinical problems caused by *S. suis*, no vaccination program against this pathogen and free of Porcine Reproductive and Respiratory Syndrome virus. All animals were probably already colonized by *S. suis* or *S. suis*-like microorganisms as they are part of the normal microbiota of the upper respiratory tract. Upon arrival, piglets were weighed, individually tagged, assigned to two groups (placebo or vaccinated; *n* = 10 per group) with equal average weight (approximately 5–6 kg), and placed in the Level II experimental animal facility of the Faculty of Veterinary Medicine, University of Montreal. Piglets were fed commercial, pelleted non-medicated food, with an addition of dry veggie supplements. The same procedure was performed for all 6 adjuvant groups of piglets for a total of 120 piglets.

### Experimental design: immunization and challenge of pigs

Two days upon arrival, piglets were immunized intramuscularly (IM) in the neck muscle, with 1 mL of formalin-killed *S. suis* serotype 2 strain P1/7 with selected adjuvant (vaccine group) or adjuvant only in PBS (placebo control group). The second dose of vaccine and placebo were administered IM two weeks after the first dose (Additional file 1). Twelve days after the second injection, the immunized and control animals were weighed, sedated using a dose of 0.5 mg/kg Atravet (Boehringer Ingelheim, Burlington, ON, Canada), and challenged with an intraperitoneal (IP) injection of 5 mL (5 × 10^9^ CFU) of a log-phase culture of *S. suis* serotype 2 strain P1/7. The average weight of the piglets on the day of the challenge was 14 kg. Blood samples were collected from the jugular vein before each immunization and before challenge for the determination of antibody responses (see below). Following the challenge, pigs were monitored three times per day over a period of nine days for the presence of clinical signs and mortality. The individuals observing the animals were blinded to the treatments. A daily clinical score was calculated based on a clinical observation sheet. Assessed were general behavior, locomotion (musculoskeletal alterations) and functional alteration of the central nervous system (CNS). Behavior clinical scores were given as follows: 0 = normal attitude and response to stimuli; 1 = slight depression with marginally delay in the response to the stimuli, but preserved appetite; 2 = moderate depression, animal only responds to repeated stimuli, reluctant to move, decreased appetite; 3 = severe depression, non-responsive, recumbent, incoordination, diminished appetite. Locomotion clinical scores were given as follows: 0 = normal gait and posture; 1 = one joint affected, light lameness, but rises and moves without assistance; 2 = moderate lameness, 2–3 joints affected with the swelling but stands without assistance; 3 = severe lameness, ataxia 3–4 joints affected, recumbent and cannot stand or move. Finally, central nervous system (CNS) clinical scores were given as follows: 0 = normal physiological behavior and response to stimuli; 1 = slight incoordination, strabismus; 2 = moderate incoordination, trembling; 3 = sever, lateral or dorsal head inclination, ataxia, opisthotonus, nystagmus, convulsions. Pigs having a clinical score = 3 in either category and a body temperature above 40 °C for two consecutive days were humanely euthanized. Ketamine (20 mg/kg; Narketan®, Vetoquinol, Lavaltrie, QC, Canada) and xylazine (2 mg/kg; Rompun®, Bayer, Mississauga ON, Canada) were administered IM to achieve complete anesthesia followed by intracardiac administration of pentobarbital sodium (100 mg/kg; Euthanyl®, Vetoquinol). Blood was collected from each piglet before euthanasia for bacteriological analyses. A post-mortem examination procedure was conducted for all pigs. Swabs were collected from meninges and synovial fluid from affected joint cavities and seeded on blood agar for bacterial recovery. Samples of liver and spleen were collected and cultured for bacterial recovery. The individuals performing the necropsies and bacterial recovery were blinded to the treatments. Samples for bacterial isolation and serotyping were taken from all euthanized animals as well as survival animals at the end of the trial.

### Measurement of antibodies against *S. suis* serotype 2 and CPS

Blood was aseptically collected from the jugular vein at three time points: before the first dose (day 0), before the second dose (day 14), and before the challenge (day 26). Blood was centrifuged 3000 × *g* for 10 min, and sera were collected and stored at −20 °C until further analysis. Sera from vaccinated and control piglets were analyzed by an indirect ELISA for antibodies against whole *S. suis* bacteria, standardized using the challenge strain, as previously reported [13]. Briefly, Polysorb plates (Nunc-Immuno; Thermo Scientific, Mississauga, ON, Canada) were coated with 100 μL/well of a suspension equivalent to 10^8^ CFU/mL *S. suis* serotype 2 strain P1/7 in ddH_2_O. Plates were air-dried for two days at room temperature (RT) and finally fixed with 50 μL/well of 100% methanol. After evaporation of methanol, plates were stored at RT until use. For ELISA, plates were washed with PBS-tween (PBS-T) containing 0.05% Tween-20 and blocked with 2% skim milk for 1 h at RT. To establish the antibody titers, pig sera were serially diluted (twofold) in PBS-T (starting with a dilution of 1/200) and incubated for 1 h at RT. For titration of pig total Ig [IgM + IgG] or IgM, plates were incubated with peroxidase-conjugated goat anti-pig total Ig [IgM + IgG] (Jackson ImmunoResearch, West Grove, PA, USA) or IgM (BioRad, Hercules, CA, USA) antibodies for 1 h at RT. For porcine IgG1 or IgG2 detection, mouse anti-porcine IgG1 or IgG2 (BioRad) was added for 1 h at RT. After washing, peroxidase-conjugated goat anti-mouse IgG (Jackson ImmunoResearch) was added for 1 h at RT. Plates were developed with 3,3′,5,5′-tetramethylbenzidine (TMB; Invitrogen, Burlington, ON, Canada) substrate, and the enzyme reaction was stopped by the addition of 0.5 M H_2_SO_4_. Absorbance was read at 450 nm with an ELISA plate reader. The reciprocal of the last serum dilution that resulted in an optical density at 450 nm (OD_450_) of ≤ 0.2 (cutoff) was considered the titer of that serum. To control inter-plate variations, an internal reference positive control was added to each plate [13]. Reaction in TMB was stopped when an OD_450_ of 1.0 was obtained for the positive internal control. Optimal dilutions of the positive internal control sera and anti-porcine antibodies or conjugates were determined during preliminary standardizations.

For selected time points, to measure anti-CPS specific antibodies, a previously developed protocol was applied [17] using native *S. suis* serotype 2 CPS purified as described [18]. Serum antibody titers were determined as described above.

### Statistical analysis

ELISA data were log-10 transformed to normalize distributions. Unless otherwise specified, a linear mixed model was used with sampling time as the within-subject fixed effect, group (vaccinated or placebo) as the between-subject fixed effect and animal identification (id) as a random effect. Piglet id was used for serology analyses. The model also took into account unequal variances in the two groups. A priori contrasts was performed to compare pairs of means adjusting the alpha level downward for each comparison with the sequential Benjamini–Hochberg procedure. In the analysis of IgG1 and IgG2 subclasses or anti-CPS antibodies in piglet sera, an equal variance *t*-test was used to compare means according to status. Survival rates were evaluated with chi-square analysis using the Kaplan–Meier method, and the significance of the difference was tested using the log-rank test. The clinical scores were transferred by ranking, and the significance of the difference between groups was determined by the *t-*test. Statistical analyses and graphing were performed using GraphPad Prism 8 software (GraphPad Software, San Diego, CA, USA). The level of statistical significance was set at 0.05.

## Results

### Survival rates and clinical signs

The goal of our study was to compare the immunogenicity and protection of bacterin vaccines formulated with 6 different commercial adjuvants: an aluminum hydroxide gel (Alhydrogel®); an oil-in-water (O/W)/ nanoparticle dual adjuvant emulsion (Emulsigen®-D); a saponin (Quil-A®); a water-in-oil-in-water (W/O/W) mineral oil-based adjuvant emulsion (Montanide™ ISA 206 VG); a water-in-oil (W/O) mineral oil-based adjuvant emulsion (Montanide™ ISA 61 VG); and a W/O/W mineral oil-based adjuvant emulsion (Montanide™ ISA 201 ISA). Results obtained for each vaccine formulation were compared to the corresponding placebos, prepared with the same adjuvant.

**Figure 1:**
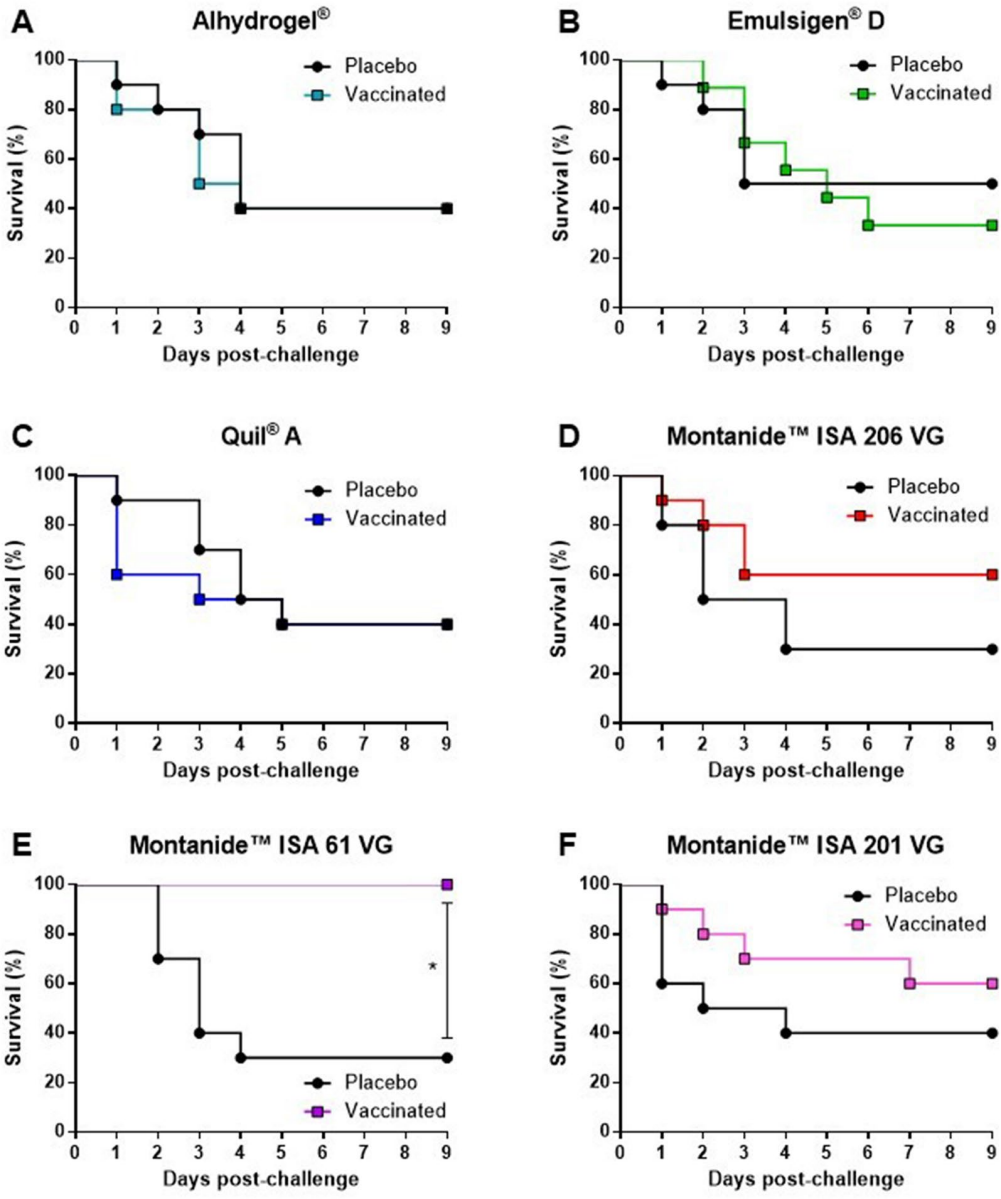
**Kaplan-Maier survival rate curves.** Piglets were vaccinated with the equivalent to 10^9^ CFU/mL of *S. suis* strain P1/7 bacterin formulated either with Alhydrogel® (**A**), Emulsigen®-D (**B**), Quil-A® (**C**), Montanide™ ISA 206 VG (**D**), Montanide™ ISA 61 VG (**E**), or Montanide™ ISA 201 VG (**F**). All piglets were challenged intraperitoneally with 5 × 10^9^ CFU of *S. suis* strain P1/7. Each vaccine formulation group of vaccinated piglets (*n* = 10) had a corresponding placebo group of piglets (*n* = 10) challenged at the same time. The clinical signs observations were conducted three times per day, nine days post-infection. *, *P* < 0.05.

**Figure 2:**
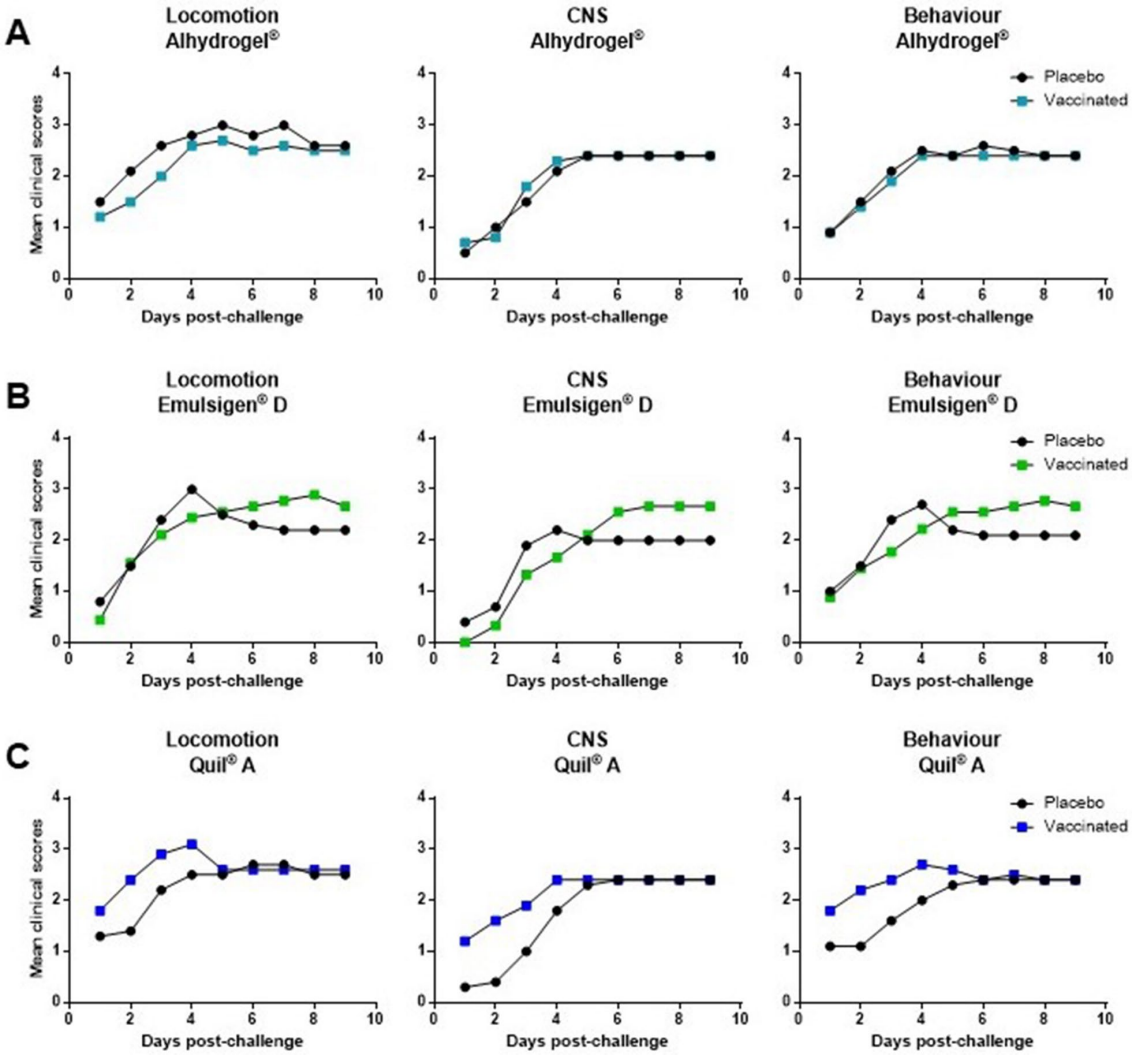
**Kaplan-Maier mean clinical scores**. Piglets were vaccinated with the equivalent to 10^9^ CFU/mL of *S. suis* strain P1/7 bacterin formulated either with Alhydrogel® (**A**), Emulsigen®-D (**B**), or Quil-A® (**C**). All piglets were challenged intraperitoneally with 5 × 10^9^ CFU of *S. suis* strain P1/7. Each vaccine formulation group of vaccinated piglets (*n* = 10) had a corresponding placebo group of piglets (*n* = 10) challenged at the same time. Clinical signs of locomotion/lameness, central nervous system (CNS), and behavior change were recorded three times per day, for nine days of the experiment, according to the scoring system explained in the materials and methods section.

**Figure 3:**
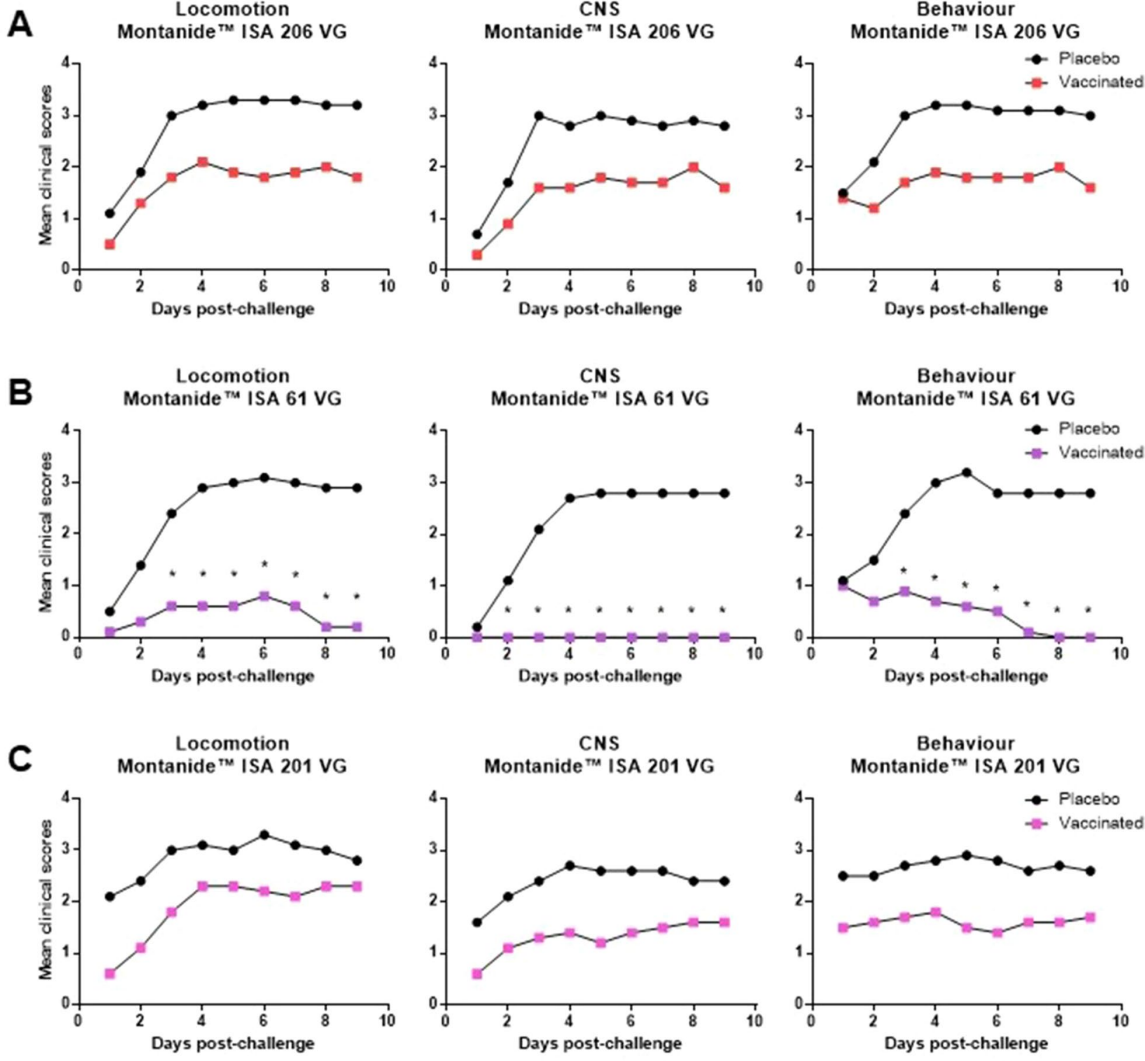
**Kaplan-Maier mean clinical scores.** Piglets were vaccinated with the equivalent to 10^9^ CFU/mL of *S*. *suis* strain P1/7 bacterin formulated with either Montanide™ ISA 206 VG (**A**), Montanide™ ISA 61 VG (**B**), or Montanide™ ISA 201 VG (**C**). All piglets were challenged intraperitoneally with 5 × 10^9^ CFU of *S. suis* strain P1/7. Each vaccine formulation group of vaccinated piglets (*n* = 10) had a corresponding placebo group of piglets (*n* = 10) challenged at the same time. Clinical signs of locomotion/lameness, central nervous system (CNS), and behavior change were recorded three times per day, for nine days of the experiment, according to the scoring system explained in the materials and methods section. *, *P* < 0.05.

The IP challenge model used in this study was able to reproduce typical clinical signs and lesions caused by *S. suis* infection in weaned piglets as those observed in the field (Additional file 2) [2]. Challenged animals showed signs of depression, incoordination, and shifting lameness (Additional file 2B). In more severe cases, there were signs of septicemia and meningitis, characterized by convulsion, head inclination, ataxia, opisthotonos, paddling, and nystagmus (Additional file 2A). Necropsy revealed typical polyarthritis lesions with abundant fibrinopurulent exudate in joint cavities (Additional file 2C). Spleen was enlarged, with petechial hemorrhages indicating systemic infection (septicemia). The IP challenge model was able to produce consistent mortality and morbidity in all six experimental placebo groups (Figures 1, 2, 3).

**Figure 4:**
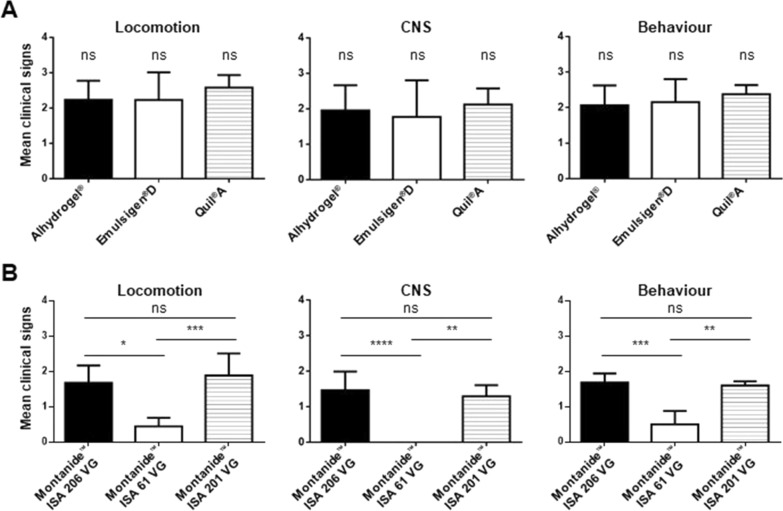
**Comparison of the mean clinical scores [locomotion, central nervous system (CNS), and behavior].** Piglets were vaccinated with bacterin vaccines formulated with Alhydrogel®, Emulsigen®-D, and Quil-A® (**A**), and piglets vaccinated with bacterin vaccines formulated with Montanide™ ISA 206 VG, Montanide™ ISA 61 VG, and Montanide™ ISA 201 VG (**B**). The error bar shows the standard deviation of the mean value of clinical scores of 10 piglets. (ns) not significant; *, *P* < 0.05; **, *P* < 0.01; ***, *P* < 0.001; ****, *P* < 0.0001.

Animals immunized with vaccines formulated with Alhydrogel® (survival rate of 40%), with Emulsigen®-D (survival rate of 33%) or with Quil-A® (survival rate of 40%) presented survival rates and clinical scores of locomotion, CNS, and behavior similar to those observed in corresponding placebos (Figures 1A–C, 2A–C). Indeed, morbidity was high in both vaccinated and placebo animals for these three adjuvant groups. Clinical signs of locomotion in a form of limping, swollen joints, and difficulties in moving, were observed in 90 to 100% of Alhydrogel®, Emulsigen®-D, and Quil-A® vaccinated piglets (Table 1). CNS clinical signs were observed in 60 to 80% of vaccinated piglets in these three adjuvant groups (Table 1) with no statistical differences between vaccinated and placebo animals. Besides, there was no statistical difference amongst these three vaccine formulations in any of the clinical sign categories (Figure 4A). Furthermore, *S. suis* was isolated from the blood, synovial fluid, and organs of vaccinated piglets at a similar rate to that of placebo animals (Table 1).

**Table 1:** **Summary of the number of piglets with clinical signs and *S. suis* isolation.**

	Locomotion	CNS/other severe clinical signs	*S. suis* in blood	Total *S. suis* isolation
*Group 1, Alhydrogel®*				
Vaccine	8/9^a^	6/10	6/8^b^	8/10
Placebo	10/10	6/10	5/8^b^	9/10
*Group 2, Emulsigen®-D*				
Vaccine	8/9^c^	7/9^c^	7/9^c^	7/9^c^
Placebo	9/9^a^	7/10	8/9^b^	9/10
*Group 3, Quil-A®*				
Vaccine	10/10	8/10	5/10	10/10
Placebo	10/10	6/10	5/10	8/10
*Group 4, Montanide™ ISA 206*				
Vaccine	7/10	6/10	4/10	6/10
Placebo	9/9^a^	9/10	7/9^b^	9/10
*Group 5, Montanide™ ISA 61*				
Vaccine	7/10	0/10	0/10	3/10
Placebo	10/10	7/10	7/10	9/10
*Group 6, Montanide™ ISA 201*				
Vaccine	8/9^a^	4/10	2/9^b^	9/10
Placebo	7/7^a^	7/10	2/7^b^	10/10

^a^ Piglets died at Day 1 thus locomotion signs could not be collected.

^b^ Blood could not be collected from one or more animals.

^c^ One piglet in the vaccine group died before the second vaccination due to causes not related to the *S. suis* infection.

The survival rate of piglets vaccinated with Montanide™ ISA 201 or 206 VG was 60%, and although the survival rates of the corresponding placebo groups were 40% and 30%, respectively, the difference was not statistically significant (Figures 1D, F). An improvement in clinical scores was observed for both vaccinated groups compared to corresponding placebos but, as in the case of mortality, there was no statistical difference during nine days (Figures 3A, C). *S. suis* was isolated in 6 out of 10 vaccinated piglets in the Montanide™ ISA 206 VG group, which is an improvement compared to 9 out of 10 animals in Montanide™ ISA 201 VG group (Table 1).

The survival rate of piglets vaccinated with Montanide™ ISA 61 VG was 100%, which was significantly different compared to the corresponding placebo with a survival rate of 30% (Figure 1E). Furthermore, clinical scores in all categories were significantly lower in the vaccinated group compared to the placebo group (Figure 3B). The vaccinated group did not have a single case of meningitis while the placebo group had 7 out of 10 animals with aggravated CNS clinical signs (Table 1). Although 7 out of 10 vaccinated animals showed clinical signs of lameness, swollen joints, and limping, the overall clinical score was low, the clinical signs were mild, and all animals were able to recover completely until the end of the trial. Only 3 out of 10 vaccinated animals had *S. suis* isolated from the joints or organs, with no isolation from the brain or blood. Opposite, *S. suis* was isolated from the joints, tissue samples, and/or blood of the majority of placebo piglets (Table 1). Finally, clinical sign scores were significantly decreased in Montanide™ ISA 61 VG vaccine group compared to the other two Montanide™ formulations (Figure 4B).

### Immunogenicity against *S. suis* serotype 2

**Figure 5:**
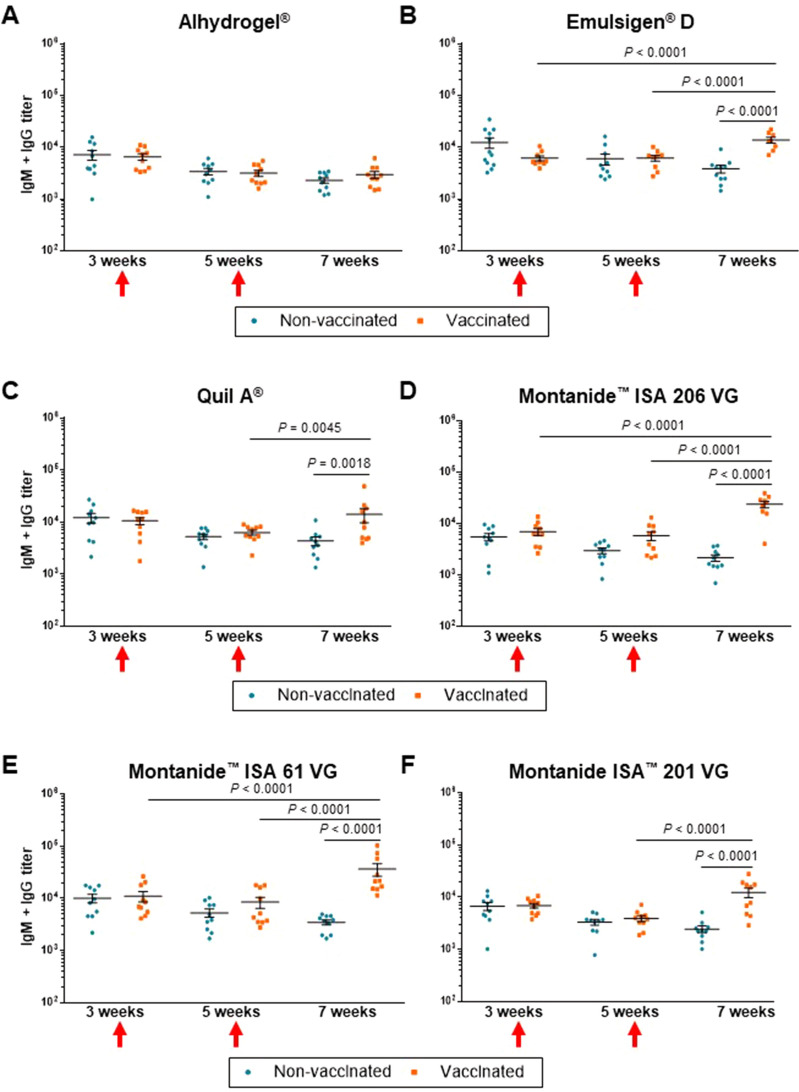
**Total Ig [IgM + IgG] levels against *S. suis* serotype 2 in piglets as determined by ELISA**. Piglets were vaccinated with the equivalent to 10^9^ CFU/mL of *S. suis* strain P1/7 bacterin formulated either with Alhydrogel® (**A**), Emulsigen®-D (**B**), Quil-A® (**C**), Montanide™ ISA 206 VG (**D**), Montanide™ ISA 61 VG (**E**), or Montanide™ ISA 201 VG (**F**). The 1^st^ dose was given at 3 weeks of age and the 2^nd^ dose at 5 weeks of age (red arrows). Each vaccine formulation group of vaccinated piglets (*n* = 10) had a corresponding placebo group of piglets (*n* = 10). Antibody titers for individual piglets are shown with horizontal bars representing mean ± SEM. Values significantly different are shown in the graph with the corresponding *P*-value.

**Figure 6:**
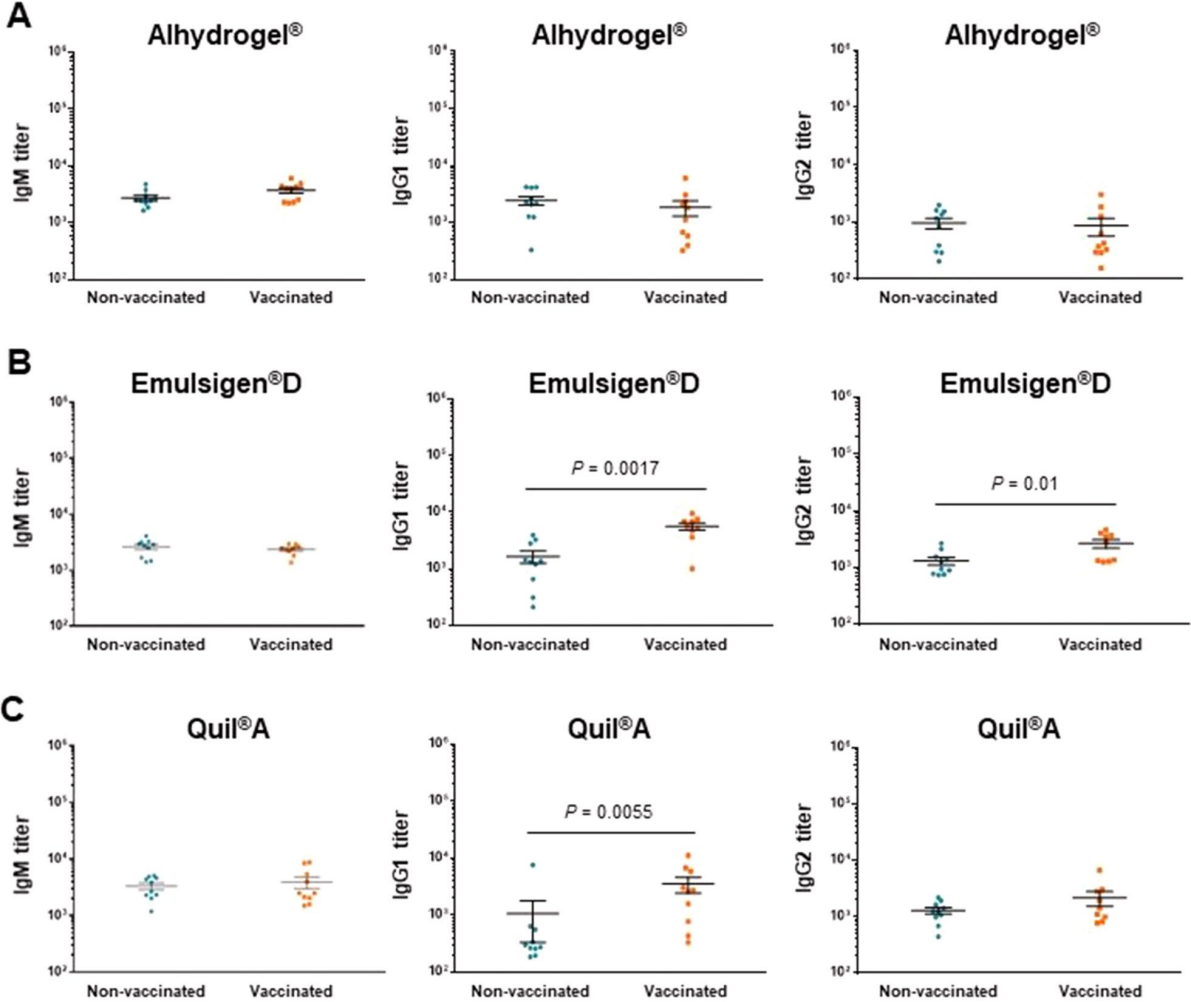
**Isotype profile of antibodies against *S. suis* serotype 2 after 2 doses of vaccine in 7-week-old piglets as determined by ELISA**. Piglets were vaccinated with the equivalent to 10^9^ CFU/mL of *S. suis* strain P1/7 bacterin formulated either with Alhydrogel® (**A**), Emulsigen®-D (**B**), or Quil-A® (**C**). The 1^st^ dose was given at 3 weeks of age and the 2^nd^ dose at 5 weeks of age. Each vaccine formulation group of vaccinated piglets (*n* = 10) had a corresponding placebo group of piglets (*n* = 10). IgM, IgG1 and IgG2 titers for individual piglets are shown with horizontal bars representing mean ± SEM. Values significantly different are shown in the graph with the corresponding *P*-value.

ELISA analyses of total Ig [IgM + IgG] antibody titers against *S. suis* serotype 2 after immunization revealed different levels of immunogenicity induced by the vaccine formulations. Upon arrival, all piglets had high basal levels of total Ig [IgM + IgG] antibody titers as detected using whole *S. suis* serotype 2 antigen, which suggests the presence of maternal antibodies acquired during the suckling period (Figure 5). These antibody levels decreased over time in all placebo groups, reaching the lowest level at 7 weeks of age (Figure 5; *P* < 0.001 vs. 3 weeks of age). Furthermore, at five weeks of age (after the 1^st^ vaccine dose), none of the vaccine formulations was able to increase the antibody titers compared to the corresponding placebos (Figure 5). Indeed, a significant increase in anti-*S. suis* antibody levels were only observed after the 2^nd^ vaccine dose for all vaccine formulations (Figures 5B–F), except for that adjuvanted with Alhydrogel® (Figure 5A). In addition, the latter vaccine formulation did not induce antibody isotype switching, since there was no difference in IgM, IgG1, and IgG2 antibody titers between Alhydrogel® placebo and vaccine group at seven weeks of age (Figure 6A).

Emulsigen®-D and Quil-A® vaccine formulations induced a significant increase in total Ig [IgM + IgG] antibody titers at seven weeks of age, after the 2^nd^ vaccine dose (Figures 5B, C). When analyzing the antibody profile (Figure 6B), it was observed that Emulsigen®-D vaccine formulation induces an increase in IgG1 (*P* = 0.001) and, to a lesser extent of IgG2 (*P* = 0.01) against *S. suis* serotype 2. On the other hand, Quil-A® vaccine formulation induced isotype switching towards IgG1 only (*P* = 0.005; Figure 6C).

**Figure 7:**
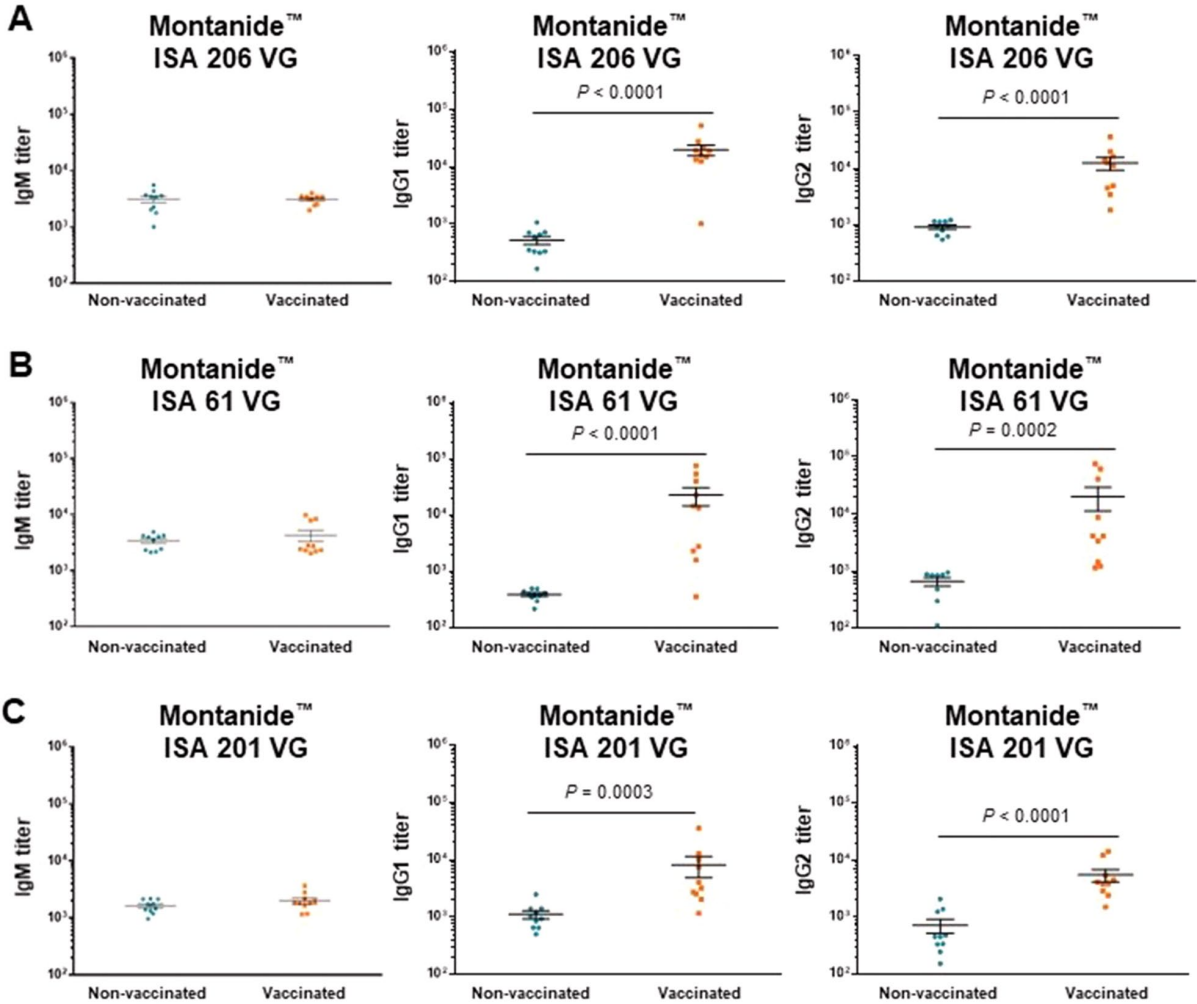
**Isotype profile of antibodies against *S. suis* serotype 2 after 2 doses of vaccine in 7-week-old piglets as determined by ELISA**. Piglets were vaccinated with the equivalent to 10^9^ CFU/mL of *S. suis* strain P1/7 bacterin formulated either Montanide™ ISA 206 VG (**A**), Montanide™ ISA 61 VG (**B**), or Montanide™ ISA 201 VG (**C**). The 1^st^ dose was given at 3 weeks of age and the 2^nd^ dose at 5 weeks of age. Each vaccine formulation group of vaccinated piglets (*n* = 10) had a corresponding placebo group of piglets (*n* = 10). IgM, IgG1 and IgG2 titers for individual piglets are shown with horizontal bars representing mean ± SEM. Values significantly different are shown in the graph with the corresponding *P*-value.

All three Montanide™ vaccine formulations were able to induce a significant increase in total Ig [IgM + IgG] antibody titers against *S. suis* serotype 2 at seven weeks of age, after the 2^nd^ vaccine dose (Figure 5D–F). The highest increase in antibody titers was observed in pigs vaccinated with the vaccine formulated Montanide™ ISA 61 VG (Figure 5E). In terms of the isotype profile of induced antibodies, a significant and marked increase in both, IgG1 and IgG2 subclasses was observed in animals immunized with the three Montanide™ vaccine formulations (Figure 7).

### Immunogenicity against *S. suis* CPS

**Figure 8:**
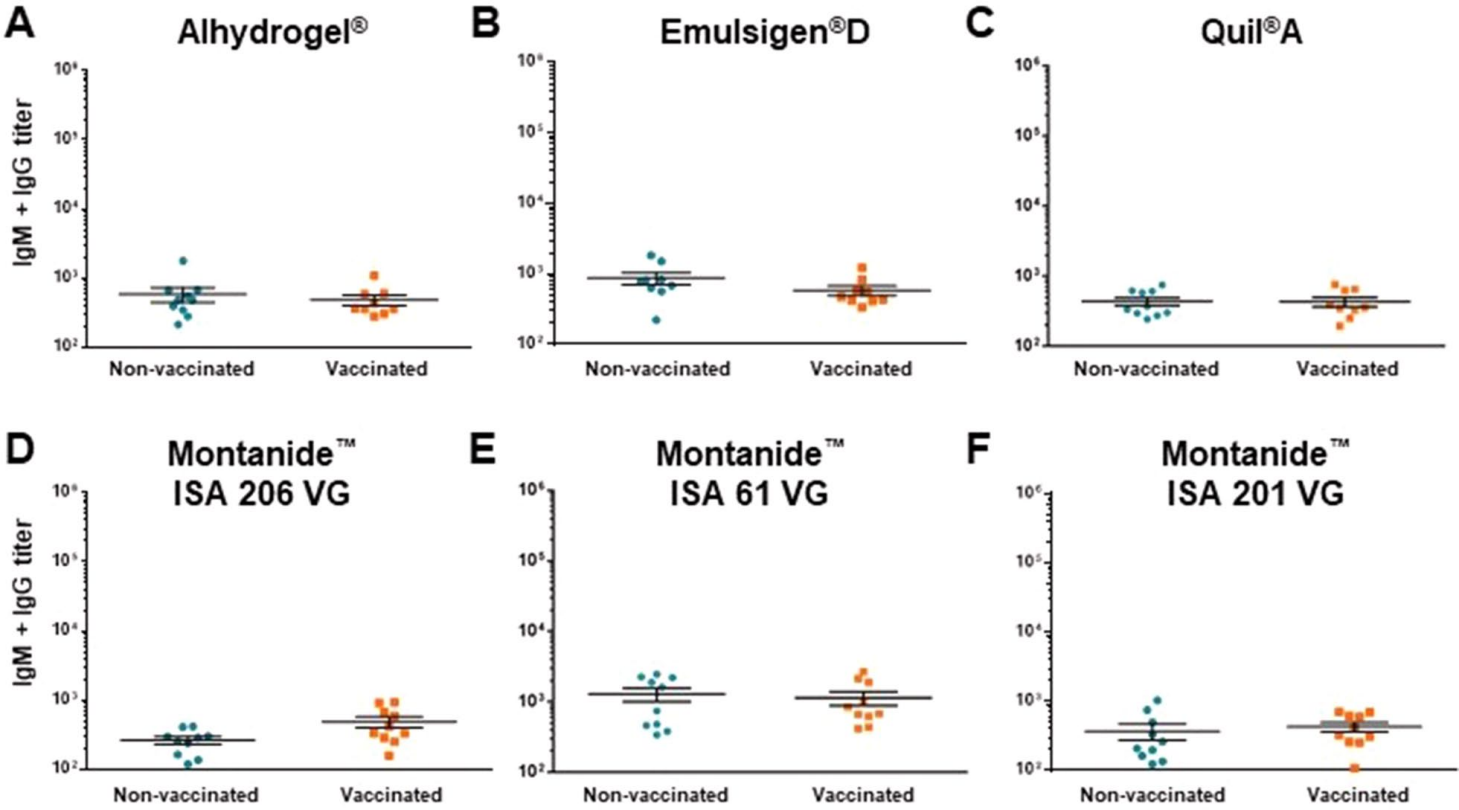
**Total Ig [IgM + IgG] levels against the purified capsular polysaccharide (CPS) of *S. suis* serotype 2 in piglets as determined by ELISA**. Piglets were vaccinated with the equivalent to 10^9^ CFU/mL of *S. suis* strain P1/7 bacterin formulated either with Alhydrogel® (**A**), Emulsigen®-D (**B**), Quil-A® (**C**), Montanide™ ISA 206 VG (**D**), Montanide™ ISA 61 VG (**E**), or Montanide™ ISA 201 VG (**F**). The 1^st^ dose was given at 3 weeks of age and the 2^nd^ dose at 5 weeks of age. Each vaccine formulation group of vaccinated piglets (*n* = 10) had a corresponding placebo group of piglets (*n* = 10). Antibody titers for individual piglets sampled at 7 weeks of age are shown with horizontal bars representing mean ± SEM.

Antibodies directed against the CPS of *S. suis* are known to be usually low but important for protection [17]. In the current study, sera from vaccinated piglets at seven weeks of age were tested to determine the effect of different vaccine formulations on the induction of CPS-specific antibodies. There was no significant increase in anti-CPS antibodies in animals immunized with none of the vaccine formulations (Figure 8).

## Discussion

Adjuvants are key components of vaccine formulations and possess multiple properties able to increase the level (magnitude) of the vaccine-induced immunological response, reduce the number of doses, control the release of the antigen (depot effect) at the site of the injection and, importantly, to modulate the type of induced immunity. The latter effect may have a major impact on the vaccine-induced protection against clinical disease [19].

Facing both the lack of effective commercial vaccines and the forthcoming restrictions in the prophylactic and metaphylactic use of antimicrobials, swine producers have increased the use of autogenous vaccines (bacterins) to prevent and control *S. suis* outbreaks. Nevertheless, as recently reviewed by Rieckmann et al. [11], there is a lack of scientific data on the actual efficacy of this type of vaccine and the immune response induced in vaccinated animals. Furthermore, only two studies have comparatively addressed the role of different adjuvants on the antibody response and/or protection induced by a *S. suis* bacterin vaccination [14, 15]. In the present work, we compared six commercial and widely used veterinary adjuvants under the same experimental conditions.

Experimental infections using virulent *S. suis* strains are not easy to reproduce [20]. In fact, presence of serious clinical signs in the field are frequently associated to co-factors, which are usually absent in research animal facilities [10]. Indeed, when using the more natural intranasal route of infection with conventional animals, the success rate on reproducing the disease is usually very low (unpublished observations). In fact, almost all animals are colonized by *S. suis* or *S. suis*-like microorganisms and it is not possible to find conventional animals “free of *S. suis*”. Caesarian-derived colostrum-deprived germ-free piglets are highly susceptible [20], but they do not represent the reality of conventional animals. Vaccination of piglets against *S. suis* aims to increase antibody-mediated bacterial killing in blood to prevent septicemia. Our systemic challenge model, using an intraperitoneal route of infection, showed high reproducibility and consistency of clinical results which is of the utmost importance when comparing the effect of adjuvants on a given vaccine formulation [20, 21]. Overall, results showed that the type of adjuvant used in the vaccine formulation has a paramount effect on the protection of piglets against *S. suis* challenge.

Maternal antibodies are known to interfere with the vaccine capacity to induce optimal immune responses, as previously recorded in immunization studies for other bacterial diseases common in piglets [22]. Vaccine interference with maternal antibodies has specifically been suggested for *S. suis* [23]. As previously reported, piglets used in the current study possessed maternal antibodies, since most sows are colonized by *S. suis* (or *S. suis*-like microorganisms) and normally possess high levels of antibodies [13, 23, 25]. High basal levels of maternal antibodies reacting with whole bacteria of *S. suis* serotype 2 were still present at weaning, which might explain the fact that one-dose immunization was not sufficient to induce a significant increase in specific anti-*S. suis* antibodies in vaccinated piglets and this independently of the adjuvant used. However, this hypothesis should still be confirmed. In the field, it is not unusual to give the first dose of vaccine at processing during the first week of life [13]. In the current study, even if the first dose of the vaccine was administered at weaning (three weeks of age), the effect was only observed after the 2^nd^ vaccine dose. The exact origin of maternal antibodies in the current study (as well as those from other studies) are unknown. In fact, the herd of origin of the animals used in this study did not have clinical signs due to *S. suis* in weaned piglets. Indeed, since whole bacteria was used as antigen for the ELISA test, it is highly possible that cross-reacting antibodies are detected. These antibodies might have been generated in adult animals by *S. suis* strains that are part of the normal microbiota or even by other *S. suis*-like microorganisms that are present in the upper respiratory tract [3, 24]. Adult animals usually present high levels of antibodies tested for any serotype of *S. suis* [13, 25] (unpublished observations), which may explain why *S. suis*-associated diseases are almost never observed in older animals [10]. Finally, in addition to the presence of some residual maternal antibody interference, other features that might also explain the lack of immunological response after one vaccine dose include an immature immune system, diet change, and other stressing factors [26, 27]. Finally, the type of antigen (*S. suis* whole encapsulated bacteria) might not be immunogenic enough to support a one-dose vaccination program.

The results of this study were able to confirm previous findings on the limited or lack of immunogenicity and/or protection of bacterin vaccines adjuvanted with aluminum hydroxide [14, 28]. This adjuvant, commonly known as alum, has been one of the most extensively used aluminum salts as an adjuvant for swine vaccines so far. It can form a short-term depot and is inexpensive, safe and simple to formulate [19]. Although it has been used for over 90 years in human and animal vaccines, there are still unknowns about the mechanisms of immune stimulation [29]. Nevertheless, our data indicate that there is no scientific rationale to use aluminum hydroxide in *S. suis* bacterin vaccine formulations due to the low immunogenicity and lack of protection in weaned piglets against *S. suis* serotype 2 experimental infection. Since the antigen can also influence the adjuvant effect and appropriate formulation, more research on the use of aluminum adjuvants for *S. suis* vaccines in swine would be required [21, 30].

As aforementioned, adjuvants have the potential to modulate the features of the antibody response induced by the vaccine. Indeed, the protection ability of the diverse Ig classes and subclasses depends on the specificity and affinity with the targeted antigen and their biological functions. In mice, IgG2b, IgG2c, and IgG3 are considered “type 1 IgG subclasses” because they are associated with IFN-γ-dominant Th1 immune responses and are particularly effective at favoring bacterial opsonophagocytosis, which is known to be required to eliminate encapsulated extracellular pathogens, such as *S. suis* [21, 31, 32]. In contrast, IgG1 (called “type 2 IgG subclass”) elicited during IL-4-dominant Th2 immune responses, usually has a less protective potential [33]. In pigs, the functionality of the different IgG subclasses has not been well characterized, in part due to the lack of appropriate reagents to differentiate the complexity of swine Ig allelic variants [34, 35]. Nevertheless, based on available reagents, swine IgG2 has been suggested to correlate with better protection against *S. suis* infection [31]; yet contradictory results do exist on the swine IgG1 vs*.* IgG2 specific contribution to protection [26].

Despite the induction of humoral immunity in vaccinated piglets, bacterin formulations with Emulsigen®-D (O/W) and Quil-A® (saponin) failed to provide clinical protection against an *S. suis* serotype 2 experimental challenge and a biased IgG1 antibody response was observed. Emulsigen® and Quil-A® adjuvants have shown strong induction of humoral immunity and protection in vaccines against other major livestock pathogens [19]. A previous study demonstrated that an *S. suis* serotype 2 bacterin adjuvanted with Emulsigen® induces protective immunity against homologous challenge. The protective effect correlated with the presence of opsonizing antibody titers against the serotype 2 strain, and a mixed IgG1/IgG2 antibody response against the muramidase-released protein (used as ELISA antigen) [36]. On the other hand, an experimental sub-unit vaccine using surface antigen one (Sao) as antigen and Emulsigen®-Plus as adjuvant induced an IgG1-dominated humoral response and did not reflect in the protection of pigs against *S. suis* serotype 2. The same antigen with another adjuvant (Quil-A®) was protective [31, 33]. Finally, a *S. suis* serotype 9 bacterin adjuvanted with Emulsigen® elicited a limited humoral antibody response and did not show an association with clinical protection [37]. Studies on Quil-A® formulated *S. suis* vaccines in swine are scarce and also revealed contradictory results [21]. For instance, as mentioned above, the Sao sub-unit vaccine formulated with Quil-A® triggered a strong IgG2 biased, opsonizing antibody response in pigs which conferred efficient protection against challenge infection with *S. suis* serotype 2 [31]. A saponin-adjuvanted bacterin delayed the appearance of the clinical signs and decreased their severity after *S. suis* challenge, although it did not have a significant effect on pig mortality [15]. It should be noted, however, that it is difficult to compare the obtained results on the immunological response amongst these studies. Several factors might influence the outcome of the study for a given vaccine formulation, including the source of the adjuvant and their formulation variations, the coating antigen used for the ELISA test and/or the ELISA procedure used to calculate antibody titers. Another option that could be further explored is the combination of Emusligen®-D with aluminum hydroxide or Quil-A®. The combination of these adjuvants could potentially increase immune response as reported in previous vaccination studies against foot-and-mouth disease in pigs [38]. The obstacle to creating a vaccine with a combination of adjuvants against diseases in swine is mainly economic; the cost–benefit of such a combination should be evaluated.

Montanide™ adjuvants are commercial W/O or W/O/W emulsions used to formulate animal vaccines against different livestock diseases [39, 40]. In cattle, inactivated vaccines against foot-and-mouth disease formulated with Montanide™ ISA 61 VG, Montanide™ ISA 201 VG, or Montanide™ 206 ISA VG, all induced a long-lived immunity; however, vaccines formulated with Montanide™ ISA 201 VG showed a more rapid onset of the immune response and the highest level of cellular immunity and a mixed IgG1/IgG2 immune response [39–41]. In our work, *S. suis* vaccines formulated with one of these three Montanide™ adjuvants also induced a mixed IgG1/IgG2 immune response. Bacterin vaccines formulated with Montanide™ ISA 201 VG or Montanide™ ISA 206 VG showed similar results characterized by partial, albeit not significant, clinical protection compared to the corresponding placebos. These results could be expected since both adjuvants are W/O/W emulsions and thus have a similar mechanism of immune stimulation. On the other hand, vaccine formulation with Montanide™ ISA 61 VG showed maximal protection and significant reduction of clinical signs against a homologous *S. suis* challenge and high antibody levels. Our results corroborate a recent study, where inactivated *Listeria monocytogenes* emulsified with Montanide™ ISA 61 VG showed increased induction of antibody titers, higher IgG2a/IgG1 ratios, and 100% protection against challenge in a murine model [42]. Studies on the effect of Montanide™ adjuvants on the efficacy of *S. suis* vaccines are scarce. A comparative study reported that a Montanide™ ISA 50 (W/O) adjuvanted *S. suis* bacterin appears to be more efficacious than a Montanide ISA 25 (O/W) formulation in delaying the onset of mortality, and decreasing clinical signs and lesions associated with *S. suis* serotype 2 challenge infection [15].

Another interesting finding of our study is that neither of the tested bacterin vaccine formulations induced an increase of antibodies against *S. suis* CPS. *S. suis* CPS is regarded as an important virulence factor that facilitates survival of the bacteria during infection, and thus an important target of protective antibodies [17, 20]. Similarly, an *S. suis* serotype 2 bacterin adjuvanted with Stimune® failed to induce anti-CPS antibodies [17]; confirming the poor immunogenicity of the CPS, event when associated with the bacterial surface [43–45]. Indeed, the ability of *S. suis* bacterins to induce this type of antibody is controversial [13, 21, 36, 46]. Therefore, the observed clinical protection after piglet vaccination with the bacterin formulated with Montanide™ ISA 61 VG could not be correlated to the presence of anti-CPS antibodies. In this case, protective antibodies are probably directed against surface-exposed bacterial proteins [21].

Taken together the results from our study indicate that the type of adjuvant formulation has paramount importance on the efficacy and protection of bacterin-based vaccines against *S. suis* serotype 2. The bacterin vaccine formulated with Montanide™ ISA 61 VG was the only formulation able to provide clear significant protection against homologous challenge and reduced morbidity. It must be noted that the IP challenge dose and route used in this model are extremely high and aggressive. The goal of this challenge model was to represent the exacerbated systemic infection, thus the level of protection provided by the vaccine formulated with Montanide™ ISA 61 VG in this experimental challenge would certainly be adequate during natural infection in the field. It should be noted that almost all piglets are early colonized by *S. suis* and it is not known how this early colonization may affect the response against a vaccine. Therefore, more studies confirming the data obtained in the current study are necessary.

## Supplementary Information

**Additional file 1. Experimental design of the study.** Experimental design of the study for evaluation of immunogenicity and protection of bacterin vaccines formulated with different adjuvants. The experimental design was consistent for all tested vaccine formulations. IP; intraperitoneal injection.

**Additional file 2. Typical clinical signs of S. suis disease observed in piglets.** Typical clinical signs of *S. suis *disease observed in piglets from the placebo Montanide™ ISA 61 VG control group. The intraperitoneal challenge model used in this experimental study was able to reproduce typical *S. suis *clinical signs of meningitis (head inclination and incoordination) (A); lameness, swollen joints (black arrow), and polyarthritis (B); and characteristic lesions of fibrinopurulent exudate in swollen joints observed during necropsy (black arrow) (C). *S. suis* serotype 2 was isolated from the joint cavities, meninges, liver and spleen of diseased animals. Pigs having a clinical score = 3 were humanely euthanized.

## Data Availability

The materials and data not presented in this manuscript are available from the corresponding author upon request.

## Abbreviations

CPS: capsular polysaccharide
CNS: central nervous system
id: identification
IM: intramuscularly
IP: intraperitoneal
O/W: oil-in-water
PBS: phosphate-buffered saline
PBS-T: PBS-tween
RT: room temperature
Sao: surface antigen one
THB: Todd–Hewitt broth
TMB: tetramethylbenzidine
W/O: water-in-oil
W/O/W: water-in-oil-in-water
