# Supplementary figures and images for: Experimental evaluation of protection and immunogenicity of Streptococcus suis bacterin-based vaccines formulated with different commercial adjuvants in weaned piglets
Source: Vet Res. 2021 Oct 19;52:133. doi: 10.1186/s13567-021-01004-x (PMC8527783; doi:10.1186/s13567-021-01004-x)

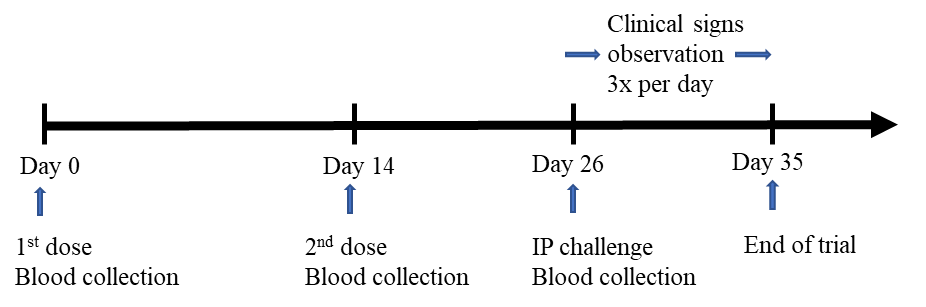

Supplement: Supplementary file 1 — Additional file 1. Experimental design of the study. Experimental design of the study for evaluation of immunogenicity and protection of bacterin vaccines formulated with different adjuvants. The experimental design was consistent for all tested vaccine formulations. IP; intraperitoneal injection. [file 13567_2021_1004_MOESM1_ESM.docx]

**C**

**B**

**A**


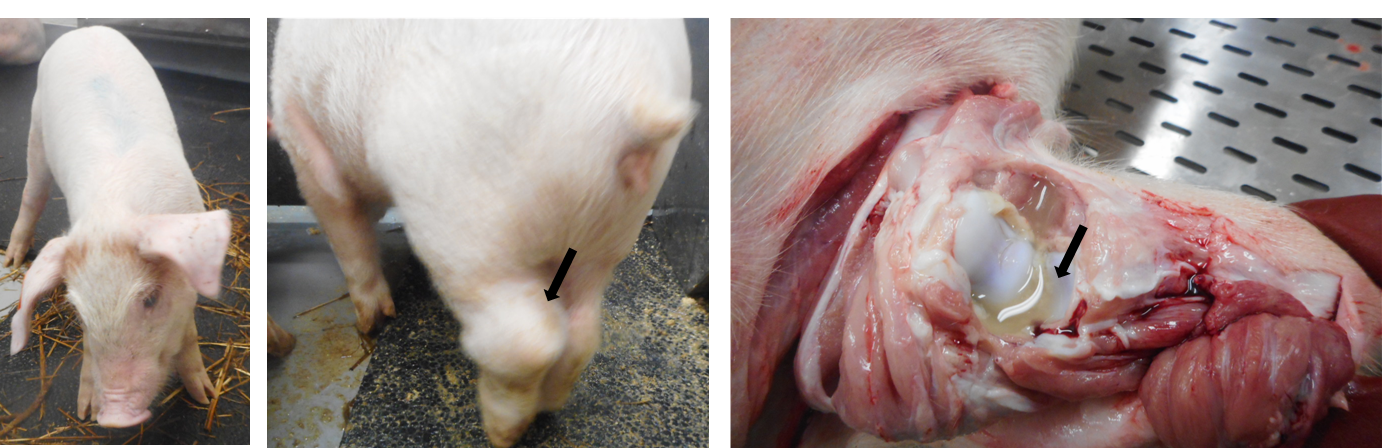

Supplement: Supplementary file 2 — Additional file 2. Typical clinical signs of S. suis disease observed in piglets. Typical clinical signs of S. suis disease observed in piglets from the placebo Montanide™ ISA 61 VG control group. The intraperitoneal challenge model used in this experimental study was able to reproduce typical S. suis clinical signs of meningitis (head inclination and incoordination) (A); lameness, swollen joints (black arrow), and polyarthritis (B); and characteristic lesions of fibrinopurulent exudate in swollen joints observed during necropsy (black arrow) (C). S. suis serotype 2 was isolated from the joint cavities, meninges, liver and spleen of diseased animals. Pigs having a clinical score = 3 were humanely euthanized. [file 13567_2021_1004_MOESM2_ESM.docx]
